# Adaptations to infer fitness interdependence promote the evolution of cooperation

**DOI:** 10.1073/pnas.2312242120

**Published:** 2023-12-06

**Authors:** Marco Colnaghi, Fernando P. Santos, Paul A. M. Van Lange, Daniel Balliet

**Affiliations:** ^a^Department of Experimental and Applied Psychology, Institute for Brain and Behaviour Amsterdam, Vrije Universiteit Amsterdam, Amsterdam 1081BT, The Netherlands; ^b^Informatics Institute, University of Amsterdam, Amsterdam 1098XH, The Netherlands

**Keywords:** cooperation, Prisoner’s Dilemma, evolutionary game theory, modeling, interdependence

## Abstract

The evolution of cooperation is a major question in the biological and behavioral sciences. While most theoretical studies model cooperation in the context of an isolated interaction (e.g., a Prisoner’s Dilemma), humans live in heterogeneous social environments, characterized by large variations in fitness interdependence—the extent to which one’s fitness is affected by others. Theoretical and experimental work indicates that humans can infer, and respond to, variations in interdependence. In a heterogeneous ancestral environment, these psychological mechanisms to infer fitness interdependence could have provided a selective advantage, allowing individuals to maximize their fitness by deciding when and with whom to cooperate. Yet, to date, the link between cognitive inference, variation in fitness interdependence, and cooperation remains unclear. Here we introduce a theoretical framework to study the evolution of inference and cooperation in heterogeneous social environments, where individuals experience interactions with varying levels of corresponding interests. Using a combination of evolutionary game theory and agent-based modeling, we model the evolution of adaptive agents, who incur a cost to infer interdependence, in populations of fixed-behavior agents who always cooperate or defect. Our results indicate that natural selection could promote the evolution of psychological mechanisms to infer fitness interdependence, provided that there is enough variation in fitness interdependence to offset the cost of inference. Under certain conditions, the fixation of adaptive agents results in higher levels of cooperation. This depends crucially on the type of inference performed and the features of the interdependence landscape.

## Significance

Much theory on the evolution of cooperation has focused on interactions described by a single payoff matrix. However, human social ecologies involve a great variety of interdependent interactions, some of which involve a conflict of interests (e.g., prisoner’s dilemma) and others that involve more corresponding interests. In heterogeneous social landscapes, a mechanism to infer interdependence could be advantageous and impact the evolution of cooperation. Here, we introduce a theoretical model to investigate the evolution of these psychological adaptations and their impact on cooperation in environments varying in conflict of interests. We found that natural selection favors the evolution of inference of fitness interdependence, especially in heterogeneous environments, promoting cooperation even when defection is the dominant strategy in the average interaction.

The evolution of cooperation, where an individual incurs a cost to provide a benefit to a recipient, has been the focus of decades of research (1–7). A number of ecological features have been shown to enhance the benefits of cooperation and make defection less advantageous, including genetic relatedness (1, 8), repeated interactions (9–11), gossip and reputation (12–14), and the structure of the social networks (15–17). These mechanisms reduce the conflict of interests within an interaction, thereby promoting the evolution of cooperation.

When interactions are simultaneous, altruistic cooperation can be modeled as a Prisoner’s Dilemma (3) (PD), a social dilemma where cooperation leads to better outcomes than mutual defection, but the best outcomes are achieved by exploiting a cooperative partner. Historically, a PD described by a single payoff matrix has been a paradigmatic approach to investigate the evolution of cooperation (4–6, 18, 19). But ecological features such as reciprocity, population structure, or reputation can change the nature of social interactions so that defection is not a dominant strategy anymore and cooperative equilibria emerge (3). For example, individuals may interact with others who have opportunities to retaliate, or within social networks where actions’ observability and shared social norms may dictate future reciprocity or punishment (e.g., ingroup versus outgroup members). Interactions are then best described as other types of social dilemma, such as Stag Hunt/Assurance (SH) or Chicken/Snowdrift game (CH), which are more favorable for cooperation (6, 20). Thus, decisions to cooperate can occur within different payoff structures across a lifetime, which could be characterized in terms of different degrees of corresponding versus conflicting interests.

Indeed, humans (and many other animals) experience a complex and heterogeneous social landscape—an “ecology of games” (21)—with wide variations in fitness interdependence between individuals (i.e., the extent to which an individual’s survival and reproduction is positively or negatively affected by others (22, 23). As a consequence, an individual typically experiences varying levels of corresponding interests depending on the social environment where they are embedded (24). For example, the same interaction could be a prisoner’s dilemma, a stag hunt, or a maximizing difference game depending on whether one’s social partner (or third parties) can retaliate or adapt their behaviors in future encounters. Likewise, even in one-shot interactions, small differences in the environment can fundamentally change the interdependence of an interaction: for example, a third-party observer of an interaction who can gossip to others in their network, thereby affecting the actors’ reputations, can make non-cooperation a less tempting option.

Variability in fitness interdependence could have created a selection pressure for psychological mechanisms to infer fitness interdependence (22, 25, 26). Experimental studies show that humans can infer variation in corresponding interests across social interactions (25–27) and, importantly, adapt their behavior accordingly (28–30). Psychological mechanisms to infer fitness interdependence across interactions could have provided a selective advantage in an ancestral environment characterized by variability in corresponding interests, allowing individuals to maximize their fitness by appropriately deciding when and with whom to cooperate. In such environment, there could have been selective pressure for the ability to integrate information about relatedness, likelihood of repeated encounters, reputation, and other cues of fitness interdependence, in order to estimate the degree of corresponding versus conflicting interests within an interaction and modulate one’s behavior accordingly (25, 29). Yet, to date, it remains unclear which conditions can promote the evolution of a psychological mechanism to infer fitness interdependence, and whether this can in turn favor the emergence of cooperation in heterogeneous interdependence landscapes.

Recent theoretical work demonstrated that heterogeneity in fitness interdependence in the form of stochastic games (31, 32), multiple games (33, 34), and games with fluctuating payoffs (35, 36) facilitates the emergence of cooperation. In structured populations where individuals play a PD game with their neighbors, adding a perturbation term to the payoff matrix can lead to the formation of clusters of cooperators (35, 36). Similarly, the alternation between different games or the presence of games with multiple states (stochastic games) can lead to the emergence of cooperative equilibria even when defection is the dominant strategy in individual games (31, 32, 34). Bear and Rand (33) investigated the evolution of the ability to discriminate between situations that favor high or low cooperation by examining differences between iterated and one-shot PD. If the probability of repeated interaction is above a certain threshold, natural selection favors the spread of adaptive cooperators, which can discriminate between the two types of interaction and modulate their behavior accordingly. In this model, cooperators who can infer the type of interaction outperform defectors even when defection is the best strategy in the average game.

Despite these theoretical advances, the role of inferring fitness interdependence in the evolution of cooperation is still poorly understood. With the notable exception of Bear and Rand (33), most theoretical efforts have been focused on strategies that are conditional on a partner’s behavior [e.g., tit-for-tat (9)] or on the outcome of a previous interaction [e.g., win-stay-lose-shift (10)], rather than on the ability to respond to the features of the interdependence structure (e.g., the degree of corresponding interests or the type of game). If ancestral social ecologies were indeed characterized by variability in fitness interdependence, then the ability to infer this feature could have provided some adaptive benefits and favored the evolution of cooperation.

Here we develop a theoretical framework to investigate the evolution of psychological mechanisms to infer fitness interdependence. In our theoretical model, agents engage in a range of different interactions during their lifetimes, with payoff matrices drawn from a random distribution. We assume that variation around an average game is due to different features which can impact fitness interdependence, such as the likelihood of repeated interaction, direct and indirect reciprocity, and population structure. Therefore, our model does not rely on any explicit assumptions about the specific mechanisms that cause this variation, as in real populations heterogeneity is likely to arise from a multitude of different sources.

**Fig. 1.**
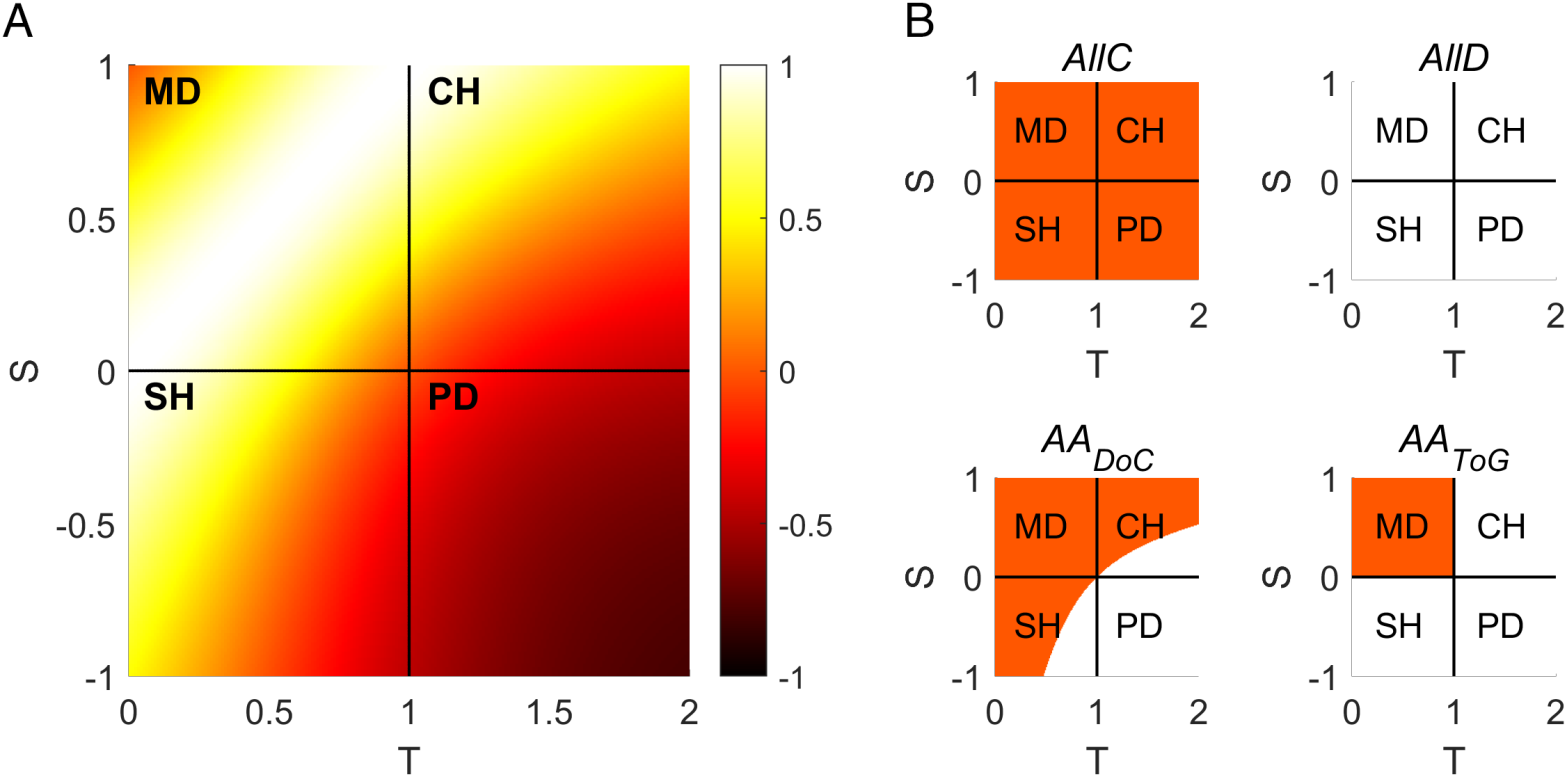
Variation in degree of corresponding interests and type of games (*A*) and visual representation of different strategies (*B*). (*A*) Changes in S and T lead to four possible games: MD ( S>0  , T<1  ), CH ( S>0  , T>1  ), SH ( S<0  , T<1  ) and PD ( S<0  , T>1  ). The degree of correspondence of interests is highest in the MD domain and lowest in the PD domain; SH and CH are characterized by varying levels of correspondence of interest, decreasing with lower values of S  and higher values of T  . (*B*) The region of the parameter space where different types of agents cooperate (defect) is shown in orange (white). We consider two types of fixed behavior agents, *AllC* and *AllD*, who always cooperate and defect, respectively, and two types of adaptive agents: *AA_DoC_* cooperate when the degree of correspondence of an interaction is positive, *AA_ToG_*only cooperate when cooperation is the dominant strategy (i.e., in the MD domain).

We generate different distributions of payoff matrices by fixing the values of the reward for mutual cooperation ( R=1 ) and the punishment for mutual defection ( P=0 ), and sampling values of the temptation to defect ( T ) and the “sucker’s payoff” ( S ) randomly from a uniform distribution (*Materials and Methods*). By changing T and S, we create ecologies with different games [PD, CH, SH, and Maximizing Difference (MD)] and varying levels of corresponding interests (Fig. 1*A*). Importantly, individuals lack complete information about the payoffs involved in the heterogeneous set of interactions played. The fitness of an individual is proportional to the average outcome over multiple interactions. To disentangle the impact of inference from other mechanisms that are known to promote cooperation, such as population structure and repeated interactions, we assume a well-mixed population (i.e., every individual has an equal probability of interacting with any other) and one-shot interactions.

We study the dynamics of populations where fixed-behavior agents (who either always defect or always cooperate) compete with adaptive agents, whose decision to cooperate or defect is based on their (costly) knowledge of the interdependence structure for each interaction (Fig. 1*B*). While agents do not possess full information about payoffs, adaptive agents pay a cost to infer some key features of interdependence. Prior research has forwarded two models of how humans infer interdependence (37), involving either the degree of correspondence of interests of an interaction (25, 29) or the classification of the type of game – that is, whether a social interaction is best described as a PD, SH, CH, or MD game (27). Thus, we consider two kinds of adaptive agents: *AA_DoC_* (Degree of Correspondence inference), who incur a cost to infer the degree of correspondence of interests of an interaction (25, 29), and *AA_ToG_* (Type of Game inference), who incur a cost to infer the type of game (27). After inferring the degree of correspondence or the type of game, adaptive agents can employ an action tailored to the information they inferred: they will cooperate if the degree of correspondence is positive or, when inferring the type of game, when cooperation is a dominant strategy (i.e., in MD games; see Fig. 1).

Using a combination of evolutionary game theory and agent-based modeling, we evaluate what distributions of games make costly inference of fitness interdependence an evolutionarily stable strategy, under what conditions it can evolve, and whether the spread of adaptive agents can lead to higher levels of cooperation.

## Results

**Fig. 2.**
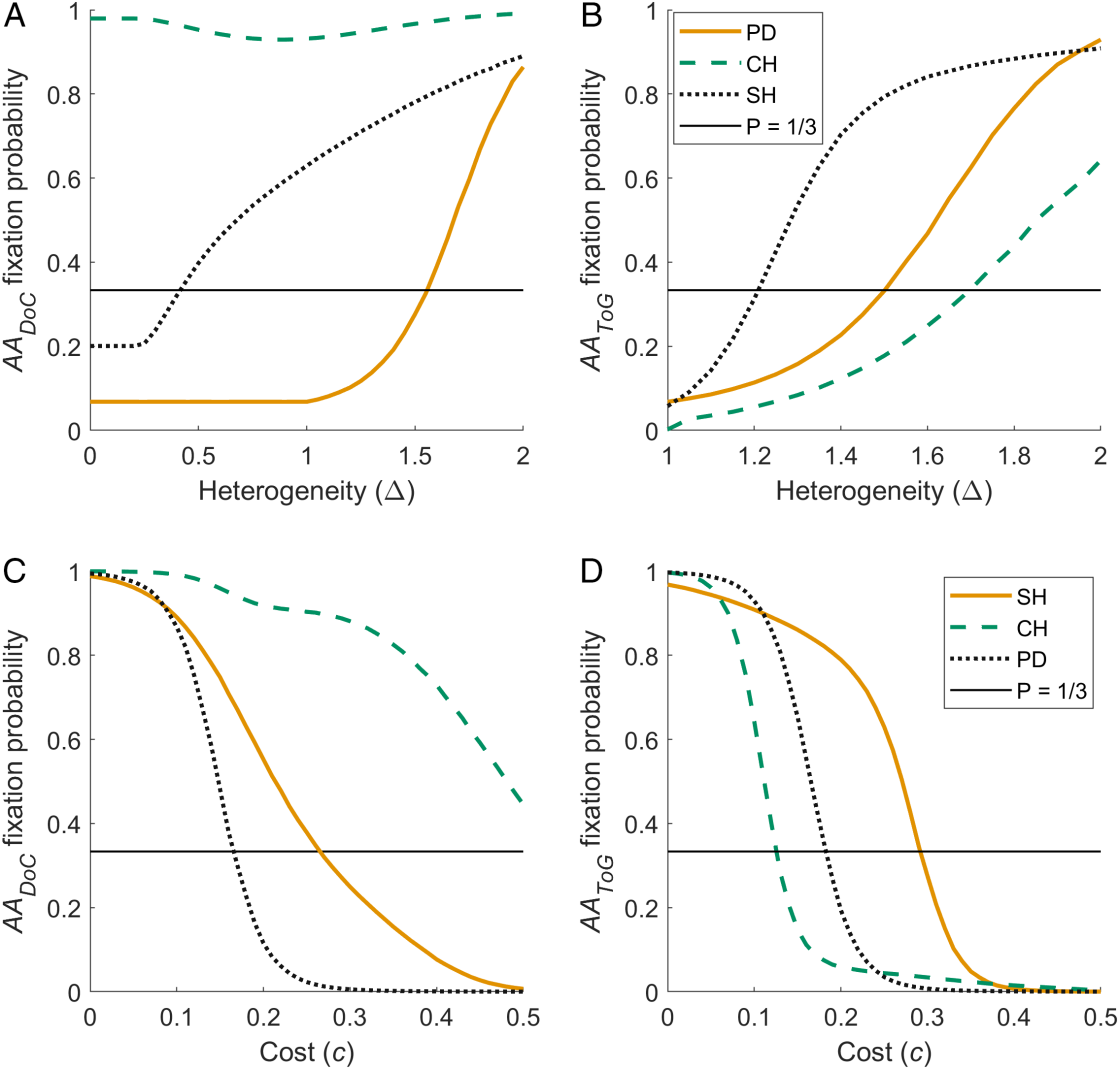
Fixation probability of adaptive agents as a function of the level of heterogeneity Δ  (*A* and *B*) and cost of inference c  (*C* and *D*). This is calculated in environments where the average game is an SH ( $\langle T\rangle =0.5,\;\langle S\rangle =-0.5$  ; in blue), a CH ( $\langle T\rangle =1.5,\;\langle S\rangle =0.5$  ; in orange), and a PD ( $\langle T\rangle =1.5,\;\langle S\rangle =-0.5$  ; in teal), respectively. (*A*) and (*C*) show the fixation probability of *AA_DoC_*, (*B*) and (*D*) the fixation probability of *AA_ToG_*. The continuous line indicates the fixation probability of a neutral mutation ( P=1/3 ). Other parameters: N=60,β=1,c=0.1 . Initial conditions: (X,Y,Z=20,20,20).

Fig. 2 shows how heterogeneity and cost affect the fixation of adaptive agents in environments where the average game is an SH ( $\langle T\rangle =0.5,\;\langle S\rangle =-0.5$  ), CH ( $\langle T\rangle =1.5,\;\langle S\rangle =0.5$  ), or PD ( $\langle T\rangle =1.5,\;\langle S\rangle =-0.5$  ). For both types of agents (degree of correspondence inference, *AA_DoC_*, shown in the left plots, and type of game inference, *AA_ToG_*, shown in the right plots), the probability of fixation increases in more heterogeneous environments (i.e., higher Δ ; Fig. 2 *A*–*B*). This observation holds true in all types of environments, with the only exception of *AA_ToG_* agents in SH environments, where fixation probability is high even when heterogeneity is low. The probability of fixation of both types of agents declines as the cost of inference ( c ) increases (Fig. 2 *C* and *D*). When the cost of inference is low, adaptive agents dominate fixed-behavior strategies (*AllC* and *AllD*), reaching fixation with a higher probability than the neutral expectation. As the cost of inference increases, the probability of fixation of adaptive agents declines. How quickly fixation probability declines as inference becomes more costly depends on the type of agents and the distribution of games considered; for example, *DoC* inference is beneficial in CH environments even when the cost is high ( c=0.5 ), while *AA_ToG_* can afford more costly inference in SH environments.

**Fig. 3.**
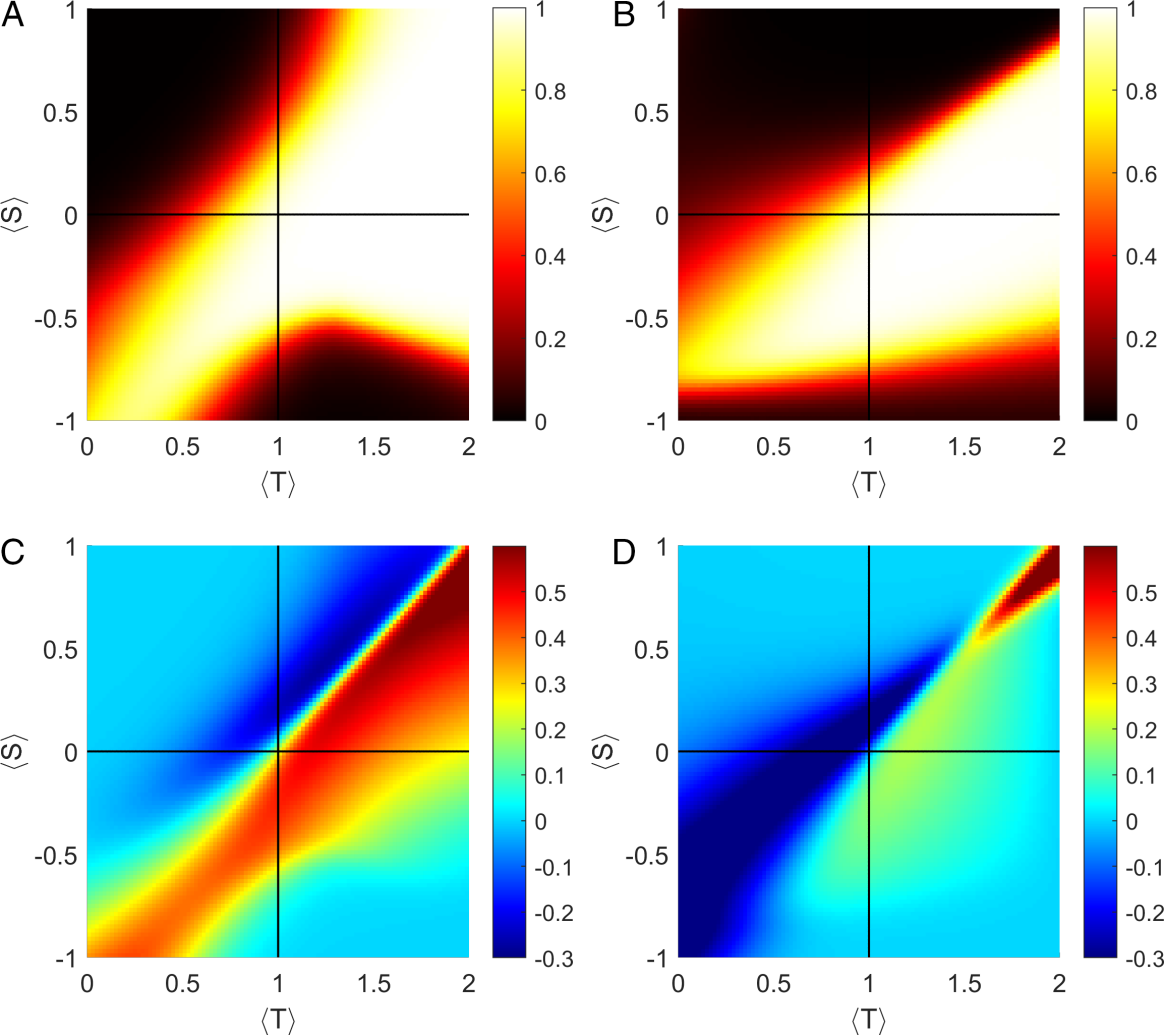
(*A* and *B*) Fixation probability of adaptive agents who pay a cost c to infer the degree of correspondence of an interaction, cooperating in games with non-negative degree of corresponding interests (*A*) or the type of game, cooperating in games where cooperation is the dominant strategy (*B*). (*C* and *D*) Increase in average cooperation levels (compared to those of fixed-behavior agents only) in the presence of adaptive agents who can infer either the degree of corresponding interests (*C*) or the type of game (*D*). Parameters: N=60,Δ=2,β=1,c=0.1 . Initial conditions: (X,Y,Z=20,20,20).

We next considered how different distributions of games influence the fixation probability of adaptive strategies (Fig. 3 *A* and *B*) and the level of cooperation (Fig. 3 *C* and *D*) in a population where adaptive agents, cooperators, and defectors are equally represented. When the average game is an MD ( T<1  , S>0  ), neither adaptive strategy is particularly advantageous, although inference of the type of game (*AA_ToG_*) is slightly more beneficial than the degree of correspondence inference (*AA_DoC_*) (Fig. 3 *A* and *B*). In the MD domain, the introduction of *AA_DoC_* does not markedly affect the level of cooperation (Fig. 3*C*), while the introduction of *AA_ToG_* is either neutral or decreases cooperation (Fig. 3*D*). When the average game is CH ( T>1,S>0  ), both strategies are favored by natural selection (Fig. 3 *A* and *B*) and can either enhance or decrease cooperation. The increase in cooperation is substantial for *AA_DoC_* in a large region of the CH domain, and moderate for *AA_ToG_* (Fig. 3 *C* and *D*). In this domain, both strategies can also lead to a decrease in cooperation (Fig. 3 *C* and *D*). While both adaptive strategies are favored by natural selection in a wide range of SH ecologies ( T<1,S<0  ) (Fig. 3 *A* and *B*), they have opposite effects on the level cooperation: Specifically, cooperation increases in the presence of *AA_DoC_*, but declines with the presence of *AA_ToG_* (Fig. 3 *C* and *D*). When the average game is PD ( T>1 , S<0 ), both types of agents are favored in a large region of the parameter space (Fig. 3 *A* and *B*) and enhance the level of cooperation. However, in the PD domain *AA_DoC_* increase cooperation to a greater extent than *AA_ToG_* (Fig. 3 *C* and *D*). Overall, *AA_DoC_* are favored by natural selection in a larger region (65.30% of the parameter space) than *AA_ToG_* (55.56%), and their presence generally enhances the level of cooperation (Fig. 3*C*). The impact of *AA_ToG_* on cooperation is more ambivalent: in a large area of the parameter space (especially in the PD domain), *AA_ToG_* mildly enhance cooperation; in another region (especially in the SH domain), they markedly reduce cooperation (Fig. 3*D*). To evaluate the robustness of these results, we also calculated the fixation probability of a small number of adaptive agents in populations largely composed of fixed-behavior agents (*SI Appendix*, Fig. S2). This analysis yielded similar results, showing that adaptive agents reach fixation with a probability higher than the neutral expectation in the same regions of the parameter space (*SI Appendix*, Fig. S2 *C* and *D*).

**Fig. 4.**
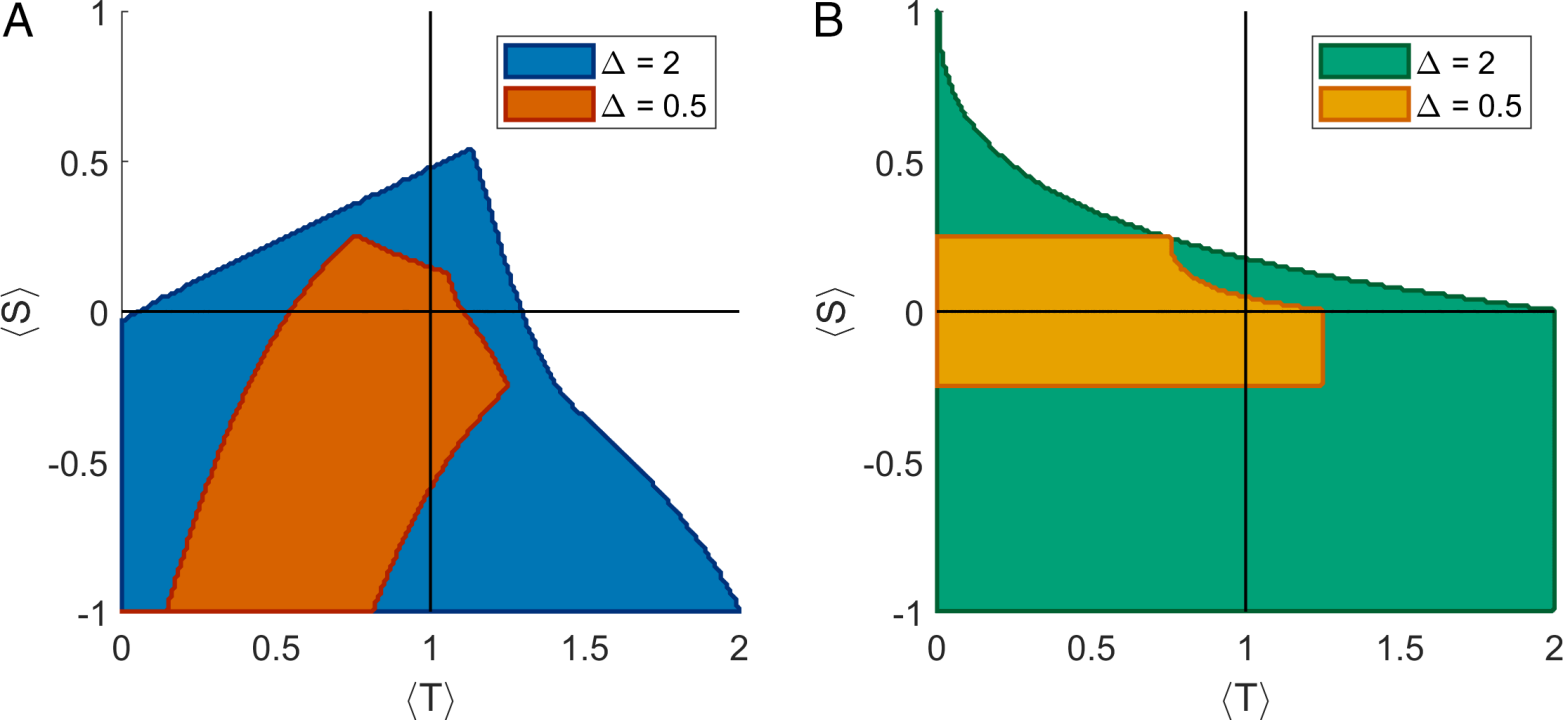
Regions of the parameter space where inference of the degree of correspondence of an interaction (*A*) or the type of game (*B*) is an ESS in highly heterogeneous ( Δ=2 ) and highly homogeneous ( Δ=0.5 ) distributions of games. Higher heterogeneity in games increases the stability of adaptive agents. In highly heterogeneous environments ( Δ=2 ), type-of-game inference is an ESS in a wider area of the parameter space than degree-of-correspondence inference.

Using a standard game-theoretical approach, we evaluated the region of the parameter space where adaptive strategies are stable against the invasion by fixed-behavior strategies for different levels of Δ . We found that higher heterogeneity in the distribution of games ( Δ=2 ) results in greater stability of both types of adaptive agents (Fig. 4). In ecologies characterized by low heterogeneity in the distribution of games ( Δ=0.1 ), *AA_DoC_* and *AA_ToG_* are an evolutionarily stable strategy (ESS) in a small region of the SH domain and across the border between the MD and SH domains, respectively (Fig. 4). As heterogeneity in games increases, the region of the parameter space where inference is an ESS becomes broader, expanding into the SH, PD, and MD domains (Fig. 4). In highly heterogeneous ecologies ( Δ=2 ), DoC inference is an ESS in the whole SH domain, more than half of the PD domain, and parts of the MD and CH domains (49.13% of the parameter space). ToG inference is stable in the whole SH and PD domains and parts of the MD and CH domains (60.64% of the parameter space), a wider area of the parameter space than DoC (Fig. 4).

## Discussion

Recent work has stressed that humans experience variability in (fitness) interdependence across their lifetime (23, 27), and that natural selection could favor psychological mechanisms to infer several features of interdependence, such as the degree of conflicting interests (25, 29). In heterogeneous social ecologies, inference of fitness interdependence could allow an individual to maximize her fitness by adopting the optimal behavior in each social interaction. Yet, most work on the evolution of cooperation assumes that all interactions can be described using a single payoff matrix, i.e., that there is no variation in the levels of fitness interdependence across individuals (1–5, 19). In the present work, we examined whether heterogeneity in fitness interdependence could create selection pressure for the agents’ ability to infer key features of social interactions and adjust their behavior according to the output of these inferences.

Our work indicates that heterogeneous social ecologies can promote the evolution of inference of fitness interdependence and cooperation, even when defection is the dominant strategy in the average game. Using a combination of evolutionary game theory and agent-based modeling, we investigated the evolutionary dynamics of two types of adaptive agents, who pay a cost to infer either the degree of corresponding interests of an interaction (*AA_DoC_*) or the type of game (*AA_ToG_*; i.e., whether an interaction is a PD, SH, MD, or CH), and decide whether to defect or cooperate based on this information: *AA_DoC_* cooperate when the degree of correspondence is non-negative ( DoC≥0  ), *AA_ToG_*when cooperation is a dominant strategy of the inferred game (Fig. 1). These two types of adaptive agents reflect previous experimental work about how people regard interdependence (25, 27, 29). We compared the success of these adaptive agents against two types of fixed-behavior agents, who either always cooperate or always defect (Fig. 1).

We found that heterogeneous social ecologies (i.e., distributions of games) promote the spread of adaptive agents, provided that there is enough variation in fitness interdependence to offset the cost of inference (Fig. 2). When heterogeneity is high ( Δ=1,2 ), adaptive strategies have a high probability of reaching fixation, even when the average game is a PD and if the cost of inference is a considerable fraction of the maximum payoff. By contrast, when heterogeneity is low ( Δ=0.1 ), inference can only evolve if its cost is negligible (Fig. 2 and *SI Appendix*, Fig. S1). Besides the level of heterogeneity, the trade-off between benefits and costs of adaptations to infer fitness interdependence changes depending on the features of the average game.

In heterogeneous social environments ( Δ=2 ), both types of inference (*AA_DoC_* and *AA_ToG_*) provide a selective advantage in a considerable region of the parameter space (Fig. 3 *A* and *B*), which is slightly greater in the case of *AA_DoC_*. These two strategies, however, influence the level of cooperation differently. The ability to infer the degree of correspondence tends to noticeably enhance the levels of cooperation across a large region of the parameter space (Fig. 3*C*), while inferring the type of game can either promote or reduce cooperation, depending on the type of ecology considered (Fig. 3*D*). More specifically, *ToG* inference lowers cooperation in a large area of the SH domain and parts of the MD and CH domains, while increasing cooperation across the PD domain and in parts of the CH domain (but to a lesser extent than *AA_DoC_*). This is likely due to the fact that *AA_ToG_* are less cooperative than *AA_DoC_*. Increasing the value of Δ (i.e., considering more heterogeneous distributions of games) widens the region of the parameter space where inference is an Evolutionarily Stable Strategy (ESS) against invasion by fixed-behavior agents (always cooperate or always defect; Fig. 4). At high Δ , this area is wider for *AA_ToG_* than *AA_DoC_* inference: while *AA_DoC_* are more likely to reach fixation, *AA_ToG_* is less likely to fix, but once it does, it is stable in a wider region of the parameter space. These results hold true when the initial number of adaptive agents in a population is minimal (*SI Appendix*, Fig. S2).

Our models do not explicitly define the source of variation in fitness interdependence, and use variation around an average game to implicitly model the impact of different ecological factors. From a quantitative perspective, the net effect of features such as repeated encounters, reputation, or punishment opportunities, is to change the structure of the payoff matrix representing a given interaction. For example, an iterated prisoner’s dilemma between reciprocal cooperators (i.e., tit-for-tat) and defectors (always defect) is equivalent to a one-shot stag hunt or a maximizing difference game, if the probability of repeated interaction is high enough (3, 33). Consistent with these claims, empirical research has shown that the expectation of repeated interaction increases cooperation in social dilemmas (38). Similarly, factors such as population structure (e.g., a more densely interconnected social network) can transform a prisoner’s dilemma into a stag hunt or a maximizing difference game (3). Our work is agnostic with regards to the source of heterogeneity, as real populations are likely to experience variation in fitness interdependence due to the combination of multiple factors.

Previous modeling work has examined the impact of heterogeneity on the evolution of cooperation (33, 35, 36, 39, 40). In ecologies where individual behavior is decoupled from the specific features of an interaction (i.e., if strategies are independent from games) and the population is unstructured, heterogeneity has a negligible impact on evolutionary dynamics, leading to the same global behavior found in a population playing the average game (40). These conditions, however, are rarely satisfied in nature, as many real populations are structured (41, 42) and the decision to cooperate or defect is often conditional on features of the interaction [at least in humans (27, 37, 43, 44)]. In structured populations, heterogeneity (in the form of payoff fluctuations) can promote the evolution of cooperation even when interactions would, on average, promote defection as the dominant strategy (35, 36, 39). When strategy is conditional on the type of game, heterogeneity has been shown to promote the evolution of agents who can discriminate between different types of interaction and modulate their behavior accordingly (33). Using a model where individuals experience either one-shot or repeated interactions with a certain probability, Bear and Rand (33) showed how natural selection favors the evolution of cooperative agents who occasionally pay a cost to discriminate between the two. While broadly consistent with the results outlined above, our approach integrates and complements them, providing a unifying framework to study the evolution of inference in highly complex ecologies of interdependence.

Our work concludes that evolution could select for psychological mechanisms to infer fitness interdependence and that this could promote cooperation within a population. A large body of experimental work supports the idea that humans can infer the degree of corresponding versus conflicting interests in an interaction and use this information to decide whether or not to cooperate (25, 26, 29). People are able to infer whether an interaction in daily life has corresponding versus conflicting interests (29), people agree in their evaluations about the extent to which an interaction involves conflicting interests (24), and that people are more likely to cooperate within interactions they perceive as having stronger corresponding interests (27, 37, 44). In fact, PD experiments that contain greater conflicting interests elicit less cooperation compared to PD experiments with less conflicting interests (43). Humans may also be able to classify interactions according to four “archetypal” games: MD, where cooperation is always beneficial; SH, where the optimal strategy is to imitate one’s partner choice; CH, where the optimal strategy is to do the opposite of one’s partner; and PD, where defection is the dominant strategy (27). Thus, future research is necessary to test different models of how humans infer their interdependence with others (37).

Understanding how and why interdependence varies can shed light on the kind of psychological mechanisms which evolved to infer interdependence. Our approach was directed by two models of variation in interdependence: 1) interdependence theory, which claims interdependence varies along several dimensions, and 2) a model of four archetypal games that are proxies for a broader range of social interactions (27, 45). Yet, there exist alternative methodologies to model variation in interdependence, for example by creating taxonomies of games (46). While future work may consider different approaches to model variation in interdependence, our current analysis does include the four games that have been most extensively studied in the theoretical and experimental research on cooperation (27, 35, 47). Moreover, the degree of correspondence of interests can be calculated for any payoff matrix, regardless of its classification (26).

For simplicity, we modeled strategies that were “hardwired,” i.e., that did not evolve over time. Allowing inference rules themselves to evolve could provide additional insights into the emergence of cooperative behavior. For example, adaptive agents could learn to cooperate more as the frequency of cooperators in a population increases (i.e., increase their level of trust that one’s partner will cooperate), leading to even higher levels of cooperation once they reach fixation. In addition, our model did not consider the possibility of error when inferring fitness interdependence: agents always infer the correct value of the degree of correspondence or the correct type of game. Error could be built into the model by including a random noise term to the inference process. This term could be inversely proportional to the cost of inference, and future studies could investigate the tradeoffs between a cheap, but noisy, and a costly, but precise, inference of fitness interdependence.

The conclusions of the present study could be broadened by investigating the impact of other forms of variation in fitness interdependence, such as asymmetric dependence (i.e., power) (25, 26). Our model only considered symmetric interactions, where both agents share the same level of power in determining their own and other’s fitness outcomes. This is obviously not the case among humans and other animals, and it has been suggested that power asymmetries can destabilize social dilemmas, making it harder for cooperation to emerge (48). The evolution of inference in more diverse ecologies of games, with a specific focus on asymmetric interactions, is a topic deserving further theoretical attention.

Our work indicates that heterogeneity in fitness interdependence can create a selection pressure for adaptations to infer interdependence. Such adaptations could either infer the degree of corresponding versus conflicting interests, or of the game structure (e.g., PD, SH, CH, or MD). Additional information about the distribution of games encountered in an ecology would help to determine whether inferring the type of game is more or less advantageous than inferring the degree of corresponding interests. Being able to infer fitness interdependence could enable strategies that allow individuals to act in their best interests and, as we show here, could elevate levels of cooperation within a population. Future research is necessary to understand the psychological mechanisms underlying the inference of interdependence and how these inferences can regulate key features of cooperation, such as who to select as a partner, when to cooperate, and how to evaluate and respond to others’ behaviors.

## Materials and Methods

### Heterogeneity in Fitness Interdependence.

The outcomes of an interaction between two agents who can either defect or cooperate can be represented using a payoff matrix of the form $g=\left(\begin{array}{cc} R & S\\ T & P \end{array}\right)$  , where R  is the reward for mutual cooperation, T  is the temptation to defect, P  is the punishment for mutual defection, and S  is the sucker’s payoff” of a cooperator who interacts with a defector. In line with previous theoretical studies (35, 36, 39, 47), we assume that mutual cooperation is always more advantageous than mutual defection, and fix the values of R and P to the arbitrary values of 1 and 0 . We introduce heterogeneity in fitness interdependence by varying the parameters S and T , and consider a distribution of games $G=\left\{g_{1},\ldots ,g_{m}\right\}$ where each interaction is described by payoff matrices of the following form:

[1] $$g_{i}=\left(\begin{array}{cc} 1 & S_{i}\\ T_{i} & 0 \end{array}\right)=\left(\begin{array}{cc} 1 & S_{0}+\epsilon_{i}^{S}\\ T_{0}+\epsilon_{i}^{T} & 0 \end{array}\right),$$

where $S_{0}$  and $T_{0}$  are the sucker’s payoff and the temptation to defect of the average game in a given ecology, and $\epsilon_{i}^{S}$  and $\epsilon_{i}^{T}$  are random variables drawn from the uniform distribution on the interval [-Δ/2,+Δ/2]  . Increasing the value of Δ  , the width of the interval from which $S_{i}$  and $T_{i}$  are sampled, leads to a higher level of heterogeneity; the broader the interval, the greater the variance in fitness interdependence. Different values of $S_{i}$  and $T_{i}$  correspond to four possible archetypal games: Prisoner’s Dilemma (PD; $T_{i}>1$  , $S_{i}<0$  ), Stag Hunt (SH; $T_{i}<1$  , $S_{i}<0$  ), Chicken/Trust Game (CH; $T_{i}>1$  , $S_{i}>0$  ), and Maximizing Difference/Harmony Game (MD; $T_{i}<0$  , $S_{i}>0$  ) (27, 35, 39).

### Types of Agents.

A central feature of our model is the presence of adaptive agents, who pay a cost ( c  ) to infer key features of a social interactions. We consider two types of adaptive agents. *AA_DoC_* (Degree of Correspondence inference agents) pay a cost c  to infer the degree of correspondence of interests of an interaction (described by a payoff matrix $g_{i}$  ) and cooperate if the degree of correspondence is non-negative, i.e., $D\left(g_{i}\right)\geq 0$  [see *SI Appendix*, *Degree of Correspondence* for detailed calculations (25, 29)]. This type of agent approximates the behavior of humans, which cooperate with a higher probability in interactions they perceive as having stronger corresponding interests, and are less likely to cooperate when conflict of interest is stronger (27, 37, 43, 44). *AA_ToG_* (Type of Game inference agents) pay a cost c to infer the type of game, that is, whether an interaction is a Prisoner’s Dilemma (PD), a Stag Hunt (SH), a Chicken Game (CG), or Maximizing Difference (MD) game (27). *AA_ToG_* only cooperate when cooperation is the dominant strategy of a given interaction, that is, only in MD games ( $R_{i}>T_{i}$ , $S_{i}>P_{i}$).

We also consider two types of fixed-behavior agents: always cooperate (*AllC*) and always defect (*AllD*). In all mathematical and computational models introduced here, these two types of fixed-behavior agents compete with each other and with one kind of adaptive agent (either *AA_DoC_* or *AA_ToG_*).

### Finite Population: Agent-Based Evolutionary Model.

To evaluate under what conditions inference of fitness interdependence can spread to fixation, we model the evolution a finite and well-mixed population of N agents. We assume that agents experience a large number of interactions during their lifetimes, so that their fitness is determined by the average payoff across the whole ecology of games. We model the evolutionary dynamics of the population using a pairwise comparison process with a Fermi probability distribution (49). At each time-step, two agents, k and j , are randomly selected from the population and k replaces j with probability:

$$p=\frac{1}{1+e^{-\beta \left(\pi_{k}-\pi_{j}\right)}},$$

where $\pi_{k}$ and $\pi_{j}$ are the average payoffs (i.e., fitness) of agent k and j (detailed calculations of the average payoffs can be found in *SI Appendix*, *Payoff Calculations in Finite Populations*). The parameter β controls the intensity of selection: for β=0 , the outcome of social interactions has no bearing on fitness (i.e., the probability that the first agent replaces the other is exactly one half), while for β→∞ the first agent replaces the second only if her fitness is higher, corresponding to strong selection (49). In our work, we assume moderately strong selection (i.e., β=1 ). The qualitative conclusions of our study do not depend on the specific choice of β.

We model the evolution of a population where two types of fixed-behavior agents, always cooperate (*AllC*) and always defect (*AllD*), compete with each other and with one kind of adaptive agent (either *AA_DoC_* or *AA_ToG_*). Each possible state of the population is described by a triplet $\left(X,Y,Z\right)$  , where X  , Y  , and Z  are the number of adaptive agents, *AllC*, and *AllD*, respectively. As an initial condition, we consider a state where the three strategies (*AllC*, *AllD*, and *AA_DoC_*/*AA_ToG_*) are present in equal proportions. To evaluate the robustness of our results, we also analyze the fixation probability of adaptive agents when they are a small minority of the population, using the state ( N/2-1,N/2-1,2 ) as an initial condition. We describe the evolution of the population as a stochastic process (Birth-death process modeled as an absorbing Markov chain) with transition probabilities defined in *SI Appendix*, *Transition Probabilities*.

The system has three absorbing states: *AA* fixation $\left(N,0,0\right)$  , *AllC* fixation $\left(0,N,0\right)$  , and *AllD* fixation $\left(0,0,N\right)$  . Using the formalism described in *SI Appendix*, *Absorption Probabilities*, we calculate the probability of reaching these three states starting from an initial state where all strategies are present in equal proportion $\left(N/3,N/3,N/3\right)$ . This allows us to evaluate the fixation probability of adaptive agents starting from an unbiased state, where all strategies are equally represented (this analysis is complemented by the subsequent analysis of evolutionarily stable strategies, which does not rely on the choice of a specific initial state). Let $\phi_{A}$ and $\phi_{C}$ be the probability of *AA* and *AllC* fixation, respectively. If adaptive agents cooperate with a frequency α (which depends on the type of adaptive agent and the distribution of games), the average expected level of cooperation is given by the fixation probability of *AllC* plus the fixation probability of *AA* multiplied by α:

$$\langle P_{c}\rangle =\phi_{C}+\alpha \phi_{A}.$$

This value expresses the probability that, after the system has reached equilibrium, a randomly observed interaction is cooperative.

### Infinite Population: Evolutionarily Stable Strategies.

We employ a standard evolutionary game theoretical approach to evaluate when inference of fitness interdependence is stable against the invasion by fixed-behavior agents (*AllC* and *AllD*). Given a distribution of games $G=\left\{g_{1},\ldots ,g_{m}\right\}$  , let $\alpha^{+}$  be the frequency of games with non-negative degree of correspondence. The payoffs of *AA_DoC_*, *AllC*, and *AllD* in a population where *AA_DoC_* have reached fixation are given by:

$$\pi \left(AA_{DoC},AA_{DoC}\right)=\alpha^{+}-c,$$

$$\pi \left(AllC,AA_{DoC}\right)=\alpha^{+}+{\langle S\rangle }^{-},$$

$$\pi \left(AllD,AA_{DoC}\right)={\langle T\rangle }^{+},$$

where ${\langle x\rangle }^{+}=\sum_{i=1}^{m}x_{i}H\left(D\left(g_{i}\right)\right)/\alpha_{\mathrm{DoC}}$ and ${〈x〉}^{-}=\sum_{i=1}^{m}x_{i}H\left(-D\left(g_{i}\right)\right)/$$\left(1-\alpha_{\mathrm{DoC}}\right)$ are the average value of the variable x across all interactions with non-negative and negative degree of correspondence, respectively, and H(x) is the Heaviside function. The payoffs of *AA_DoC_* in a population of *AllC* or *AllD* are given by $\pi \left(AA_{DoC,}AllC\right)=\alpha^{+}+{\langle T\rangle }^{-}$ and $\pi \left(AA_{DoC},AllD\right)={\langle S\rangle }^{+}$ , while the payoffs of resident strategies are $\pi \left(AllC,AllC\right)=1$ and $\pi \left(AllD,AllD\right)=0$ , respectively.

Using Eq. **1**, we sample $m=10^{6}$ payoff matrices with S and T drawn at random from the uniform distribution on the intervals $\left[S_{0}-\Delta /2,S_{0}+\Delta /2\right]$ and $\left[T_{0}-\Delta /2,T_{0}+\Delta /2\right]$ and computed the payoffs of resident and invading strategies. For different $\left(S_{0},T_{0}\right)$ pairs, we evaluate whether *AA_DoC_* satisfies the requirements of an Evolutionarily Stable Strategy (ESS): either $\pi \left(AA_{DoC},AA_{DoC}\right)>\pi \left(x*,AA_{DoC}\right)$ , or $\pi \left(AA_{DoC},AA_{DoC}\right)=\pi \left(x*,AA_{DoC}\right)$ and $\pi \left(AA_{Doc,}x*\right)>\pi \left(x*,x*\right)$.

Similarly, the payoffs in a population where *AA_ToG_* have reached fixation can be calculated by substituting $\alpha^{+}$ in the equations above with $\alpha^{\mathrm{MD}}$ , the frequency of MD games, and by calculating the averages over all MD or non-MD games:

$$\pi \left(AA_{ToG},AA_{ToG}\right)=\alpha^{\mathrm{MD}}-c,$$

$$\pi \left(AllC,AA_{ToG}\right)=\alpha^{\mathrm{MD}}+{\langle S\rangle }^{non-\mathrm{MD}},$$

$$\pi \left(AllD,AA_{ToG}\right)={\langle T\rangle }^{\mathrm{MD}},$$

where ${\langle x\rangle }^{\mathrm{MD}}=\sum_{i=1}^{m}x_{i}H\left(\mathrm{MD}\left(g_{i}\right)\right)/\alpha_{\mathrm{MD}}$ and $\begin{array}{c} {〈x〉}^{non-\mathrm{MD}}=\sum_{i=1}^{m}x_{i} \end{array}$ $H\left(-\mathrm{MD}\left(g_{i}\right)\right)/\left(1-\alpha_{\mathrm{MD}}\right)$ , where $\mathrm{MD}\left(g_{i}\right)=1$ if $g_{i}$ is a MD game, and 0 otherwise. As in the previous case, we evaluated whether *AA_ToG_* is an ESS for different values of $\left(S_{0},T_{0}\right)$.

## Supplementary Material

Appendix 01 (PDF)

## Data Availability

MATLAB code data have been deposited in GitHub (https://github.com/MColnaghi/interdependence-inference-cooperation) (50).

## References

[1] W. D. Hamilton, The genetical evolution of social behaviour II. J. Theoret. Biol. **7**, 17–52 (1964). 5875340 10.1016/0022-5193(64)90039-6

[2] J. Maynard-Smith, Evolution and the Theory of Games (Cambridge University Press, 1982).

[3] M. A. Nowak, Five rules for the evolution of cooperation. Science **314**, 1560–1563 (2006). 17158317 10.1126/science.1133755 PMC3279745

[4] R. Axelrod, The Evolution of Cooperation (Basic Books, 1984).

[5] K. Sigmund, The Calculus of Selfishness (Princeton University Press, 2010).

[6] B. Skyrms, The Stag Hunt and the Evolution of Social Structure (Cambridge University Press, 2004).

[7] P. A. M. Van Lange, D. G. Rand, Human cooperation and the crises of climate change, COVID-19, and misinformation. Annu. Rev. Psychol. **73**, 379–402 (2021). 34339612 10.1146/annurev-psych-020821-110044

[8] A. Gardner, A. S. Griffin, S. A. West, “Theory of cooperation” in Encyclopedia of Life Sciences, H. Kehrer-Sawatzki, Eds. (John Wiley Sons Ltd., Wiley, 2016).

[9] R. Axelrod, W. D. Hamilton, The evolution of cooperation. Science **211**, 1390–1396 (1981). 7466396 10.1126/science.7466396

[10] M. Nowak, K. A. Sigmund, A strategy of win-stay, lose-shift that outperforms tit-for-tat in the Prisoner’s Dilemma game. Nature **364**, 56–58 (1993). 8316296 10.1038/364056a0

[11] R. L. Trivers, The evolution of reciprocal altruism. Q. Rev. Biol. **46**, 35–57 (1971).

[12] M. A. Nowak, K. Sigmund, Evolution of indirect reciprocity by image scoring. Nature **393**, 573–577 (1998). 9634232 10.1038/31225

[13] F. P. Santos, J. M. Pacheco, F. C. Santos, The complexity of human cooperation under indirect reciprocity. Phil. Trans. Roy. Soc. B **376**, 1838 (2021). 10.1098/rstb.2020.0291 PMC8487734 34601904

[14] L. Schmid, K. Chatterjee, C. Hilbe, M. A. Nowak, A unified framework of direct and indirect reciprocity. Nat. Hum. Behav. **5**, 1292–1302 (2021). 33986519 10.1038/s41562-021-01114-8

[15] F. C. Santos, M. D. Santos, J. M. Pacheco, Social diversity promotes the emergence of cooperation in public goods games. Nature **454**, 213–216 (2008). 18615084 10.1038/nature06940

[16] H. Ohtsuki, C. Hauert, E. Lieberman, M. A. Nowak, A simple rule for the evolution of cooperation on graphs and social networks. Nature **441**, 502–505 (2006). 16724065 10.1038/nature04605 PMC2430087

[17] E. Lieberman, C. Hauert, M. A. Nowak, Evolutionary dynamics on graphs. Nature **433**, 312–316 (2005). 15662424 10.1038/nature03204

[18] A. Rapoport, “Prisoner’s Dilemma” in Game Theory, J. Eatwell, M. Milgate, P. Newman, Eds. (Palgrave Macmillan UK, 1989), pp. 199–204.

[19] J. Hofbauer, K. Sigmund, Evolutionary Games and Population Dynamics (Cambridge University Press, 1998).

[20] J. Maynard-Smith, The evolution of behavior. Sci. Am. **239**, 176–193 (1978). 705322 10.1038/scientificamerican0978-176

[21] N. E. Long, The local community as an ecology of games. Am. J. Sociol. **3**, 251–261 (1958).

[22] A. Aktipis , Understanding cooperation through fitness interdependence. Nat. Hum. Behav. **2**, 429–431 (2018). 31097813 10.1038/s41562-018-0378-4

[23] G. Roberts, Cooperation through interdependence. Anim. Behav. **70**, 901–908 (2005).

[24] S. Columbus, C. Molho, F. Righetti, D. Balliet, Interdependence and cooperation in daily life. J Pers. Soc. Psychol. **120**, 626 (2021). 32584097 10.1037/pspi0000253

[25] D. Balliet, J. M. Tybur, P. A. M. Van Lange, Functional interdependence theory: An evolutionary account of social situations. Pers. Soc. Psychol. Rev **21**, 361–388 (2017). 27466269 10.1177/1088868316657965

[26] H. H. Kelley , An Atlas of Interpersonal Situations (Cambridge University Press, 2003).

[27] N. Halevy, E. Y. Chou, J. K. Murnighan, Mind games: The mental representation of conflict. J Pers. Soc. Psychol. **102**, 132–148 (2012). 21910551 10.1037/a0025389

[28] D. Balliet, P. A. M. Van Lange, Trust, conflict, and cooperation: A meta-analysis. Psychol. Bull. **139**, 1090–1112 (2013). 23231532 10.1037/a0030939

[29] F. H. Gerpott, D. Balliet, S. Columbus, C. Molho, R. E. de Vries, How do people think about interdependence? A multidimensional model of subjective outcome interdependence. J. Pers. Soc. Psychol. **115**, 716–742 (2018). 28872331 10.1037/pspp0000166

[30] S. Abele, G. Stasser, C. Chartier, Conflict and coordination in the provision of public goods: A conceptual analysis of continuous and step-level games. Pers. Soc. Psychol. Rev. **14**, 385–401 (2010). 20519698 10.1177/1088868310368535

[31] C. Hilbe, Š Šimsa, K. Chatterjee, M. A. Nowak, Evolution of cooperation in stochastic games. Nature **559**, 246–249 (2018). 29973718 10.1038/s41586-018-0277-x

[32] Q. Su, A. Mcavoy, L. Wang, M. A. Nowak, Evolutionary dynamics with game transitions. Proc Natl. Acad. Sci. U.S.A. **116**, 25398–25404 (2019). 31772008 10.1073/pnas.1908936116 PMC6926053

[33] A. Bear, D. G. Rand, Intuition, deliberation, and the evolution of cooperation. Proc. Natl. Acad. Sci. U.S.A. **113**, 936–941 (2016). 26755603 10.1073/pnas.1517780113 PMC4743833

[34] M. Salahshour, Interaction between games give rise to the evolution of moral norms of cooperation. PLoS Comput. Biol. **18**, e1010429 (2022). 36173936 10.1371/journal.pcbi.1010429 PMC9521931

[35] M. A. Amaral, M. A. Javarone, Heterogeneity in evolutionary games: An analysis of the risk perception. Proc R. Soc. A. **476**, 20200116 (2020). 32523420 10.1098/rspa.2020.0116 PMC7277119

[36] G. Q. Zhang, Q. B. Sun, L. Wang, Noise-induced enhancement of network reciprocity in social dilemmas. Chaos Solitons Fractals **51**, 31–35 (2013).

[37] D. Balliet, B. Lindström, Inferences about interdependence shape cooperation. Trends Cogn Sci. **27**, 583–595 (2023). 37055313 10.1016/j.tics.2023.03.003

[38] P. A. M. Van Lange, A. Klapwijk, L. M. van Munster, How the shadow of the future might promote cooperation. Group Process Intergroup Relat. **14**, 857–870 (2011).

[39] M. A. Amaral, M. A. Javarone, Strategy equilibrium in dilemma games with off-diagonal payoff perturbations. Phys. Rev. E **101**, 062309 (2020). 32688499 10.1103/PhysRevE.101.062309

[40] M. A. Amaral, J. K. L. da Silva, L. Wardil, Cooperation in two-dimensional mixed-games. J. Phys. A Math. Theor. **48**, 445002 (2015).

[41] R. M. May, Network structure and the biology of populations. Trends Ecol. Evol. **21**, 394–399 (2006). 16815438 10.1016/j.tree.2006.03.013

[42] C. D. Thomas, W. E. Kunin, The spatial structure of populations. J. Anim. Ecol. **68**, 647–657 (1999).

[43] G. Spadaro , The Cooperation Databank: Machine-readable science accelerates research synthesis. Perspect. Psychol. Sci. **17**, 1472–1489 (2022). 35580271 10.1177/17456916211053319 PMC9442633

[44] L. L. Thompson, Information exchange in negotiation. J. Exp. Soc. Psychol. **27**, 161–179 (1991).

[45] A. Rapoport, A taxonomy of 2× 2 games. General Syst. **11**, 203–2014 (1966).

[46] B. Bruns, C. Kimmich, Archetypal games generate diverse models of power, conflict, and cooperation. Ecol. Soc. **26**, 2 (2021).

[47] F. C. Santos, J. M. Pacheco, T. Lenaerts, Evolutionary dynamics of social dilemmas in structured heterogeneous populations. Proc. Natl. Acad. Sci. U.S.A. **103**, 3490–3494 (2006). 16484371 10.1073/pnas.0508201103 PMC1413882

[48] M. S. Dawkins, Do asymmetries destabilize the prisoner’s dilemma and make reciprocal altruism unlikely? Anim. Behav. **80**, 339–341 (2010).

[49] A. Traulsen, M. A. Nowak, J. M. Pacheco, Stochastic dynamics of invasion and fixation. Phys. Rev. E. Stat. Nonlin. Soft Matter Phys. **74**, 11909 (2006). 10.1103/PhysRevE.74.011909 PMC2904085 16907129

[50] M. Colnaghi, Code for “Adaptations to infer fitness interdependence promote the evolution of cooperation”. GitHub. https://github.com/MColnaghi/interdependence-inference-cooperation. Deposited 31 July 2023. 10.1073/pnas.2312242120 PMC10723045 38055736

