# Supplementary material for: Adaptations to infer fitness interdependence promote the evolution of cooperation
Source: Proc Natl Acad Sci U S A. 2023 Dec 6;120(50):e2312242120. doi: 10.1073/pnas.2312242120 (PMC10723045; doi:10.1073/pnas.2312242120)
Supplement: Supplementary file 1 — Appendix 01 (PDF) [file pnas.2312242120.sapp.pdf]

PNAS

Supporting Information for

Adaptations to infer fitness interdependence promote the evolution of cooperation

Marco Colnaghi[1,*], Fernando P. Santos[2], Paul A. M. Van Lange[1], Daniel Balliet[1,*]

[1]Department of Experimental and Applied Psychology, Institute for Brain and Behaviour Amsterdam (IBBA), Vrije Universiteit Amsterdam, Amsterdam 1081BT, The Netherlands

[2]Informatics Institute, University of Amsterdam, Amsterdam 1098XH, The Netherlands

*Corresponding authors

Emails: m.colnaghi@vu.nl (M.C.), d.p.balliet@vu.nl (D.B.)

This PDF file includes:

SI Appendix A – Payoff calculations in finite populations
SI Appendix B – Degree of correspondence
SI Appendix C – Transition probabilities
SI Appendix D – Absorption probabilities
SI References
Figures S1 to S2

Supporting text

SI Appendix A – Payoff calculations in finite populations

For each set of initial conditions (S_0, T_0), we sample $m = 10^6$ random payoff matrices of the form described by Equation (1). We assume that agents play a large number of games during their lifetimes, so that their probability of reproduction depends on the average payoff across a large number of interactions. The payoffs of AA_{DoC} against other AA_{DoC} is equal to 1 in interactions with non-negative degree of correspondence ($D(g_i) \geq 0$) and 0 otherwise. Therefore, the average payoff of AA_{DoC} against themselves is given by:

$$\langle \pi_{AA} \rangle = \sum_{i=1}^{m} H(D(g_i)) - c = \alpha^+ - c$$

Where $H(x)$ is the Heaviside function, and α^+ is the fraction of interactions with non-negative degree of correspondence. Similarly, the average payoffs $\langle \pi_{KJ} \rangle$ of a generic strategy K against a generic strategy J can be calculated as follows:

$$\langle \pi_{AC} \rangle = \sum_{i=1}^{m} H\big(D(g_i)\big) + \sum_{i=1}^{m} T_i H\big(-D(g_i)\big) - c = \alpha^+ + (1 - \alpha_{DoC})\langle T \rangle^- - c$$
$$\langle \pi_{AD} \rangle = \sum_{i=1}^{m} S_i H\big(D(g_i)\big) - c = \alpha^+ \langle S \rangle^+ - c$$
$$\langle \pi_{CA} \rangle = \sum_{i=1}^{m} H\big(D(g_i)\big) + \sum_{i=1}^{m} S_i H\big(-D(g_i)\big) = \alpha^+ + (1 - \alpha^+)\langle S \rangle^-$$
$$\langle \pi_{CC} \rangle = 1$$
$$\langle \pi_{CD} \rangle = S_0$$
$$\langle \pi_{DA} \rangle = \sum_{i=1}^{m} T_i H\big(D(g_i)\big) = \alpha^+ \langle T \rangle^+$$
$$\langle \pi_{DC} \rangle = T_0$$
$$\langle \pi_{DD} \rangle = 0$$

Where $\langle x \rangle^+ = \sum_{i=1}^{m} x_i H\big(D(g_i)\big) / \alpha^+$ and $\langle x \rangle^- = \sum_{i=1}^{m} x_i H\big(-D(g_i)\big) / (1 - \alpha^+)$ are the average value of the variable x across all interactions with non-negative and negative degree of correspondence, respectively.

When the population is in a state (X, Y, Z), the average payoff of AA_{DoC}, *AllC*, and *AllD* are, respectively:

$$\langle \pi_A \rangle = \frac{X}{(N-1)} \langle \pi_{AA} \rangle + \frac{Y}{N} \langle \pi_{AC} \rangle + \frac{Z}{N} \langle \pi_{AD} \rangle - c$$
$$= \frac{X}{(N-1)} \alpha^+ + \frac{Y}{N} (\alpha_{DoC} + (1 - \alpha^+)\langle T \rangle^-) + \frac{Z}{N} \alpha^+ \langle S \rangle^+ - \mathrm{c}$$
$$\langle \pi_C \rangle = \frac{X}{N} \langle \pi_{CA} \rangle + \frac{Y}{N-1} \langle \pi_{CC} \rangle + \frac{Z}{N} \langle \pi_{CD} \rangle = \frac{X}{N} \alpha^+ \langle S \rangle^+ + \frac{Y}{N-1} + \frac{Z}{N} S_0$$
$$\langle \pi_D \rangle = \frac{X}{N} \langle \pi_{DA} \rangle + \frac{Y}{N} \langle \pi_{DC} \rangle + \frac{Z}{N-1} \langle \pi_{DD} \rangle = \frac{X}{N} \alpha^+ \langle T \rangle^+ + \frac{Y}{N} T_0$$

The payoffs of a population of *AllC*, *AllD*, and AA_{ToG} have the same form of the above equations, with the only difference that α^+ is replaced by α^{MD} (the fraction of MD games in a given ecology), and the averages are calculated over all MD (or non-MD) games: $\langle x \rangle^{MD} = \sum_{i=1}^{m} x_i H\big(MD(g_i)\big) / \alpha^{MD}$

and $\langle x\rangle^{non-MD} = \sum_{i=1}^{m} x_i H\big(-MD(g_i)\big)/(1-\alpha^{MD})$, where $MD(g_i) = 1$ if g_i is a MD game, and 0 otherwise.

SI Appendix B – Degree of correspondence

Following Kelley et al.[26], we calculate the degree of correspondence of an interaction described by the payoff matrix $g = \begin{pmatrix} R & S \\ T & P \end{pmatrix}$ as follows. We first define the following three quantities: Actor Control (AC), the extent to which variance in an agent's outcomes is determined by her own decision to defect or cooperate, Partner Control (PC), the extent to which variance in an agent's outcomes is determined by her partner's decision, and Joint Control (JC), the extent to which variance in an agent's outcomes is influenced by interaction terms (e.g., coordination or anti-coordination). These quantities can be quantified as follows:

$$AC = \frac{R+T}{2} - \frac{S+P}{2}$$
$$PC = \frac{R+S}{2} - \frac{T+P}{2}$$
$$JC = \frac{R+P}{2} - \frac{S+T}{2}$$

The degree of correspondence of an interaction is then given by:

$$D(g) = \frac{2AC * PC + JC^2}{AC^2 + PC^2 + JC^2}$$

It follows that $-1 \le D(g) \le 1$, with negative and positive values corresponding to interactions with conflict and correspondence of interest, respectively[1,2].

SI Appendix C – Transition probabilities

Let $p_{KJ} = \frac{1}{1+e^{-\beta(\pi_K - \pi_J)}}$ be the probability that an agent playing strategy K replaces another playing strategy J. If the population is in a state (X, Y, Z) with X adaptive agents, Y *AllC*, and Z *AllD*, the transition probabilities between adjacent states are calculated as follows:

$$p(X,Y,Z \to X+1, Y-1, Z) = \frac{X}{N}\left(\frac{Y}{N-1}\right)P_{AC}$$
$$p(X,Y,Z \to X+1, Y, Z-1) = \frac{X}{N}\left(\frac{Z}{N-1}\right)P_{AD}$$
$$p(X,Y,Z \to X-1, Y+1, Z) = \frac{Y}{N}\left(\frac{X}{N-1}\right)P_{CA}$$
$$p(X,Y,Z \to X, Y+1, Z-1) = \frac{Y}{N}\left(\frac{Z}{N-1}\right)P_{CD}$$
$$p(X,Y,Z \to X-1, Y, Z+1) = \frac{Z}{N}\left(\frac{X}{N-1}\right)P_{DA}$$
$$p(X,Y,Z \to X, Y-1, Z+1) = \frac{Z}{N}\left(\frac{Y}{N-1}\right)P_{DC}$$

The probability of remaining in the same state, therefore, is given by:

$$\begin{aligned} p(X,Y,Z \to X,Y,Z) = 1 &- p(X,Y,Z \to X+1, Y-1, Z) - p(X,Y,Z \to X+1, Y, Z-1) \\ &- p(X,Y,Z \to X-1, Y+1, Z) - p(X,Y,Z \to X, Y+1, Z-1) \\ &- p(X,Y,Z \to X-1, Y, Z+1) - p(X,Y,Z \to X, Y-1, Z+1) \end{aligned}$$

Given that at each iteration of the algorithm one agent replaces another, the transition probabilities to any other state are equal to zero.

SI Appendix D – Absorption probabilities

Consider the transition matrix T whose coefficients T_{ij} are the transition probabilities from state i to state j, calculated as in **SI Appendix C**. The matrix can be rewritten in its canonic form as

$$P = \begin{bmatrix} Q & R \\ 0 & I_3 \end{bmatrix}$$

Where Q is a t-by- t matrix whose elements are the transition probability between transient states, t is the number of transient states, R is a t-by-3 matrix of the transition probabilities between a transient and an absorbing state, and I_3 the 3-by-3 identity matrix[3]. The probability of reaching an ergodic state j starting from a transient state i is given by the (i, j)-th element of the matrix $B = (I_t - Q)^{-1}R$[3]. We use this equation to calculate the probability of reaching absorbing states $(N, 0,0)$ and $(0, N, 0)$ starting from the uniform state $(N/3, N/3, N/3)$ or $(N/2 - 1, N/2 - 1, 2)$.

SI References

1. D. Balliet, J. M. Tybur, P. A. M. Van Lange, Functional interdependence theory: an evolutionary account of social situations. Pers. Soc. Psychol. Rev **21**, 361–388 (2017).
2. H. H. Kelley, et al. *An Atlas of Interpersonal Situations.* (Cambridge University Press, 2003).
3. C. M. Grinstead, J. L. Snell, *Introduction to Probability.* (American Mathematical Society, 1977).

Supplementary Figures

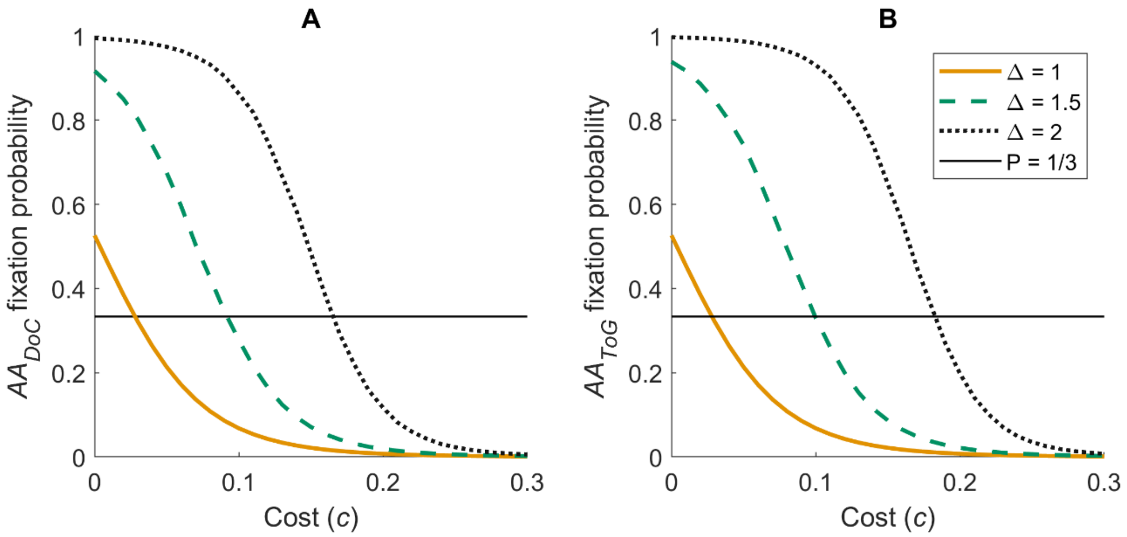

Figure S1. Fixation probability of adaptive agents who can infer the degree of correspondence of an interaction (**A**) or the type of game (**B**) as a function of the cost of inference (c), for different levels of heterogeneity in games (Δ). In more heterogeneous distributions, the benefits of inference decline more slowly with the cost, and the fixation of adaptive agents is favored by natural selection even when inference is more costly.

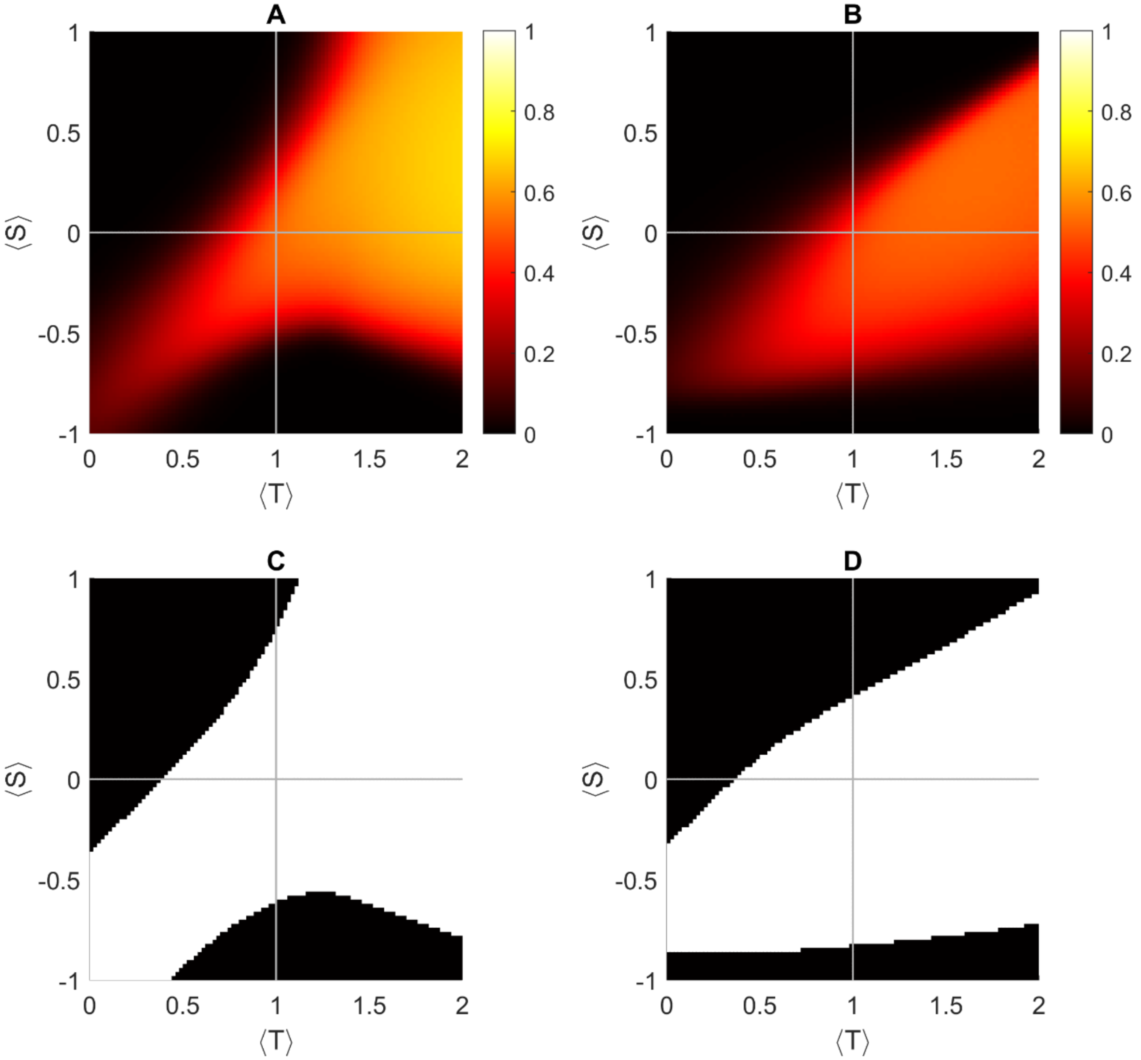

Figure S2. Fixation probability of a small number of adaptive agents in populations composed almost entirely of fixed-behavior agents. **(A-B)** Fixation probability of adaptive agents who pay a cost c to infer the degree of correspondence of an interaction, cooperating in games with non-negative degree of correspondence **(A)** or the type of game, cooperating in games where cooperation is the dominant strategy **(B)**. **(C-D)** Regions of the parameter space where the probability of fixation of adaptive agents that can infer the degree of corresponding interests (**C**) or the type of game (**D**) is greater (white) or smaller (black) than the neutral expectation (i.e., the fixation probability of a neutral adaptation, without any fitness effects). Parameters: $N = 60, \Delta = 2, \beta = 1, c = 0.1$. Initial conditions: $(X, Y, Z) = (29, 29, 2)$.
